# Annual compost amendments can replace synthetic fertilizer, improve soil moisture, and ensure tree performance during peach orchard establishment in a humid subtropical climate

**DOI:** 10.3389/fpls.2023.1172038

**Published:** 2023-05-08

**Authors:** Brian T. Lawrence, Juan Carlos Melgar

**Affiliations:** Department of Plant and Environmental Sciences, Clemson University, Clemson, SC, United States

**Keywords:** organic matter, replant, decidous fruit trees, fruit yield, fertilizer (NPK) application

## Abstract

The application of organic matter (OM) to peach orchards is currently uncommon in commercial operations but could potentially replace synthetic fertilizers and improve long-term orchard sustainability. The purpose of the study was to monitor how annual applications of compost to replace synthetic fertilizer would change soil quality, peach tree nutrient and water status, and tree performance during the first four years of orchard establishment within a subtropical climate. Food waste compost was incorporated before planting and added annually over four years with the following treatments: 1) 1x rate, applied as dry weight at 22,417 kg ha^-1^ (10 tons acre^-1^) incorporated during the first year and 11,208 kg ha^-1^ (5 tons acre^-1^) applied topically each year after; 2) 2x rate, applied as dry weight at 44,834 kg ha^-1^ (20 tons acre^-1^) incorporated during the first year and 22,417 kg ha^-1^ (10 tons acre^-1^) applied topically each year after; and 3) control, with no compost added. Treatments were applied to a virgin orchard location, where peach trees had never previously been grown, and to a replant location, where peach trees had been grown previously for more than 20 years. Synthetic fertilizer was reduced in the 1x and 2x rates by 80 and 100% during the spring and all treatments received the summer application according to standard practice. Soil OM, phosphorus and sodium all increased with the addition of 2x compost in the replant location at 15 cm depth, but not within the virgin location compared to the control treatment. The 2x rate of compost improved soil moisture during the growing season, but tree water status was similar between treatments. Tree growth was similar between treatments in the replant location, but the 2x treatment had larger trees compared to the control by the third year. Foliar nutrients were similar between treatments over the four years, while 2x compost rate increased fruit yield in the virgin location compared to the control the second year of harvest. The 2x food waste compost rate could be considered as a replacement for synthetic fertilizers and to potentially increase tree growth during orchard establishment.

## Introduction

Consumer demand for consistent and desirable quality fruits has generated a global economic and marketing system which encourages growers to primarily focus on yield, fostering orchard intensification (Losciale et al., 2020) often at the expense of orchard soils and organic matter (OM). Practices such as discing, tillage, and repeated herbicide sprays damage existing soil structure and increase erosion (Keesstra et al., 2016; Di Prima et al., 2018). Repeated and sometimes excessive use of synthetic fertilizers, herbicides, and pesticides can be harmful to the environment through leaching and runoff (Merwin et al., 1994; Brady et al., 2006; Cui et al., 2020) and accumulate over time within the soil profile when attached to soil particles (Van Bruggen et al., 2018; Aoyama & Nagumo, 1996), reducing beneficial soil microorganism populations which help create stable soil aggregates (Crouzet et al., 2019; Hale et al., 2021). Although soil quality and environmental improvement can be equally valuable economically within orchard settings for growers (Reganold et al., 2001), intensive conventional methods are still employed at the expense of beneficial agroecological functions, such as self-regulating biocontrol for pests and diseases, or the acquisition and distribution of both water and nutrients from mycorrhizal fungal networks in healthy soils (Lu et al., 2019; Granatstein, 2021). Improving orchard ecosystem services using various OM amendments has recently received renewed attention (Demestihas et al., 2017; Montanaro et al., 2017), in part due to efforts to mitigate replant disease (Mazzola & Manici, 2012; Watson et al., 2019; Diehl et al., 2020), conserve resources such as water or nutrients (Novara et al., 2021; Villa et al., 2021), and broader societal goals of carbon sequestration and sustainable development (Diacono & Montemurro, 2011; Baldi et al., 2018; Lal et al., 2021).

Different forms of OM amendments have been applied to perennial tree systems, but orchard-specific factors such as tree age (Hoagland et al., 2008), soil texture (Villa et al., 2021), application timing (Lepsch et al., 2019), sloping topography (Keesstra et al., 2019), and climate (Liu et al., 2021) can influence the benefit of a particular amendment to orchard soil health and tree water and nutritional status. Amendments which have been composted provide a superior, more stable OM source and only several years of compost application can increase soil OM, resulting in improved tree size (Moran & Schupp, 2005), soil water content, as well as tree water status (Lepsch et al., 2019). Leaf nutrient concentration after compost addition can be equal or higher to that of trees receiving synthetic fertilizers (Baldi et al., 2010b; Montanaro et al., 2012; Sorrenti et al., 2012; Baldi et al., 2016; Melo et al., 2016). Increasing OM with compost has been shown to improve yield over the first four years for apple (*Malus domestica* Borkh.) (Moran & Schupp, 2005) and over seven years for peach (*Prunus persica* (L.) Batsch) (Montanaro et al., 2012); or be equal to nectarine (*Prunus persica* cv. *nucipersica*) yield achieved with synthetic fertilizer (Baldi et al., 2018).

Despite the potential benefits that OM amendments can provide to orchards, it is uncommon for fruit growers to actively increase soil C by adding OM in subtropical regions as it can be uneconomical, and loss of OM can be rapid due to hot and humid conditions. Intensive peach production within the hot and humid region of the southeastern United States occurs primarily on Ultisols, which are low in OM from centuries of historical agricultural use (Jones et al., 2020) while microorganisms quickly break down existing OM, further limiting OM regeneration or persistence (Yue-Lin et al., 2008). Additionally, regional growers plant new trees on soil berms as a mitigation strategy against the soil pathogen Armillaria root rot (*Desarmillaria tabescens*), which often causes premature peach death on replant sites (Miller et al., 2020), increasing the frequency of soil disturbance and OM loss. Raised soil berms and ridges are also used to avoid incidence of other soil-borne diseases such as *Phytophthora* spp. for different fruit crops (Adaskaveg et al., 2008). Berms void of soil cover can increase surface water movement and channelize water on either side, increasing erosion observed in other orchards with bare soil (Keesstra et al., 2019), but the practice is preferred by growers to increase average tree life and has become the standard practice regardless of whether there is a history of the disease. New land which has lower frequency of the pathogen is often not accessible and many fruit growers establish orchards on replant sites, where legacy of prior cultivation and biological imbalances may result in replant disease (Yang et al., 2012). Adding stable compost amendments have shown promise in mitigating replant disease (Forge et al., 2016) and the creation of berms provides an opportunity for growers to incorporate soil amendments prior to tree planting. Fruit growing is also often subject to climatic variations of extreme rainfall and temperature (Eck et al., 2020) and improving soil carbon using compost may assist with production despite detrimental weather scenarios (Droste et al., 2020); for instance, peach tree growers in the southeastern U.S. often do not irrigate trees until the third year (first crop), and young trees rely exclusively on rainfall (Casamali et al., 2021). Therefore, understanding how increasing OM may improve the water status and initial growth of young trees could have an impact on orchard management.

The purpose of the study was to evaluate how annual applications of compost change soil quality, peach tree nutrient and water status, and tree performance during the first four years of orchard establishment within a subtropical climate. To be applicable to the region and grower management practices, we explored the impact of adding compost to berms and subsequently reducing synthetic fertilizer between a replant orchard and new land. The study had two broad objectives: 1) measure changes to soil properties including OM content, cation exchange capacity (CEC), nutrient content, and soil water content; and 2) measure changes to tree growth and physiology by monitoring plant size, water status, mineral nutrients, and fruit quality and yield. We hypothesized the compost-amended soils would have measurably higher OM, CEC, and nutrient content after four years and the compost treatments would increase tree biomass (trunk size and total volume), but trees would have similar mineral nutrient content. Due to larger tree size, trees planted with the 1x or 2x compost rates would have larger fruit yields, but similar fruit quality compared to the control. Trees within the composted soil would have improved tree water status during dry periods due to increased water holding capacity within the soil. Finally, we hypothesized that compost would have a more pronounced effect on tree and soil parameters examined on a replant site compared to virgin land.

## Materials and methods

### Location, design, and treatments

The study took place in two orchards at the Musser Fruit Research Center in Seneca, South Carolina (lat. 34˚36’22” N, long. 82˚52’39” W) across four growing seasons between the years of 2019 and 2022. At both locations, ‘Cresthaven’ peach trees on Guardian^®^ rootstock were planted during January 2019 at 6.7 m x 4.8 m (22 x 16 ft.) spacing and trained to an open vase pruning system. The first location was a site of a former peach orchard (replant) which had received standard orchard management (Blaauw et al., 2021) including annual synthetic fertilization application, along with numerous conventional pesticides, fungicides, and herbicide applications for over 20 years. The second orchard location, 60 m downhill from the replant location, was a site which had not previously been cultivated with peaches or any other agricultural crop for over 20 years (virgin). Before planting, rows were sprayed with herbicide (glyphosate) and later disked before adding compost and forming berms. Berms were created in both orchard locations as described by Miller et al. (2020). Throughout the study, all trees were pruned annually during dormancy, and summer pruning was also performed as needed during each growing season to ensure desired habit and prevent shading. Fruitlets were thinned by hand during years of fruit production. The compost used during the study, created from a mixture of food and yard waste, was acquired from the Clemson University Recycling Services compost facility, and varied slightly in characteristics each year of acquisition (**Table S1**).

Each orchard was divided into three treatments: 1) a low compost (1x) rate applied as dry weight at 22,417 kg ha^-1^ (10 tons acre^-1^) during year one and 11,208 kg ha^-1^ (5 tons acre^-1^) each year after; 2) a high compost (2x) rate applied as dry weight at 44,834 kg ha^-1^ (20 tons acre^-1^) during year one and 22,417 kg ha^-1^ (10 tons acre^-1^) each year after; and 3) a control rate (Control) which received no compost throughout the duration of the study. The replant orchard was planted across six rows of 8 trees each, with each treatment replicated twice in an entire row; each treatment had 16 trees. The virgin orchard was planted across six rows of 12 trees each, with the three treatments replicated once per row with four trees per treatment; each treatment had a total of 24 trees. Compost applied during year one was mixed into the soil during berm formation. Each subsequent year (2-4), compost was applied to the soil surface prior to bud burst, during late February or early March using a Millcreek row mulcher (304RM, Millcreek Mfg. Co., Honey Brook, PA, USA) and distributed using hand tools to uniformly cover berms (1 m width).

Fertilization of the control trees and pesticide/herbicide application of all treatments followed commercial guidelines throughout the study (Blaauw et al., 2021). During the first year, all trees regardless of compost treatments were fertilized the same with 67.7 kg ha^-1^ of nitrogen (N) three times between spring and summer using 10-10-10. Each year after, the rate of N was reduced in the 1x and 2x trees during the spring by 80 and 100%, respectively, when 19-19-19 was applied. Total applied nitrogen rates from synthetic fertilizer annually are listed in **Table 1**.

**Table 1:** Synthetic fertilizer spring and summer nitrogen (N, kg ha^-1^) applied during the study years by compost treatments.

Treatment	2019	2020	2021	2022
Control	67.7	67.7	67.7	67.7
1x	67.7	8.5	34.2	34.2
2x	67.7	0	25.7	25.7

Reductions of N occurred during the spring application, while no N was applied during the summer of 2020 on the 1x or 2x treatment.

### Soil parameters

Initial soil samples were taken in 2019, shortly after the treatments were applied and berms were created, with the control plots serving as a baseline to understand original soil parameters. Both orchards are classified as Cecil sandy loam (52% sand, 18% silt, and 30% clay to a depth of 15 cm, and 35% sand, 15% silt, and 50% clay to a depth of 45 cm) on land with 15 to 25% slopes and are moderately to highly eroded (Soil Survey Staff et al., 2020). Organic matter to a depth of 15 cm was found to be between 1.5 – 3% at the Musser Fruit Research Center while the bulk density of unamended soil berms was 1.15 g (cm^3^)^-1^ in the replant location and 1.01 g (cm^3^)^-1^ in the virgin location. Soil analysis of nutrients and qualities including total OM and CEC were measured during tree dormancy (February-March) annually at a depth of 15 cm (0 cm to 15 cm) and 45 cm (30 to 45 cm) measured from the top of the berm. The soil surface was cleared of any surface cover (decaying leaves) prior to taking soil samples using a 2.5 cm wide bit attached to a power drill and a 5 cm telescoping soil auger (AMS Inc., American Falls, ID, USA) for the 15 and 45 cm depth samples, respectively. A total of 4 soil samples were taken per treatment per depth within each orchard. The 15 cm depth samples were made from a composite of 8 core extractions while the 45 cm depth samples were made from a composite of 4 core extractions. Soil sample analysis included nitrate ( $NO_{3}^{-}$ ), phosphorus (P), potassium (K), calcium (Ca), magnesium (Mg), zinc (Zn), manganese (Mn), copper (Cu), boron (B), and sodium (Na) as well as pH, CEC and OM; all of which were performed by the Clemson University Agricultural Service Laboratory in Clemson, SC, USA. Soil moisture probes (Model 200SS, Irrometer Company Inc., Riverside, CA, USA) were installed to measure soil moisture tension (h_m_) every 24 hours at both 15 cm and 45 cm depths during the summer of 2019 in the replant location and during the fall of 2020 in the virgin location. Monthly averages of h_m_ were later used to make comparisons between the compost treatments in both orchard locations.

### Tree growth and stem water potential measurements

Trunk diameter measurements were taken 5 cm above the graft union to calculate the trunk cross sectional area (TCSA) during June annually. TCSA was estimated as TCSA = π*(tree diameter/2)^2^. Canopy volume was also measured beginning the second growing season during December 2020 and 2021 or October 2022 as the volume of a sphere (4/3*π*diameter^3^) using a diameter average of a horizontal measurement perpendicular to the row, and a vertical measurement from the tallest extended shoot to the bottom of the tree, excluding the distance between the lowest branches and the soil (Kusakabe et al., 2016).

Tree water status was monitored by measuring stem water potential (SWP) approximately every 3 weeks throughout the growing season beginning the second year. A total of 6 leaves were covered with foil bags to prevent light and reduce transpiration during the morning, and then later used to measure midday SWP per treatment in each orchard location using a Scholander-type pressure bomb (PMS Instrument Co., Albany, OR, USA; Scholander et al., 1965).

### Leaf nutrient analysis

Seven fully-grown tree leaves from the 4^th^ to 6^th^ node were picked annually from each tree and combined by row and treatment during the month of July for nutrient analysis. A total of six leaf samples were taken for each compost treatment within each orchard (six from the two rows in the replant location and one from each row in the virgin location). Leaf K, Ca, and Mg were measured as described by Lawrence and Melgar (2018) using atomic absorption spectrophotometry (PinAAcle 500, PerkinElmer, Waltham, MA, United States) while P was measured according to Murphy and Riley (1962) using the molybdenum blue colorimetric method. Total N was measured by combustion using a revised Dumas method (Jones et al., 1991).

### Fruit yield and analysis

Fruit yield was calculated from an average of 8 trees per compost treatment in the replant location and 12 trees per compost treatment in the virgin location in the third and fourth year of the study. Commercially ripe fruit (visually determined by background color) were harvested from the trees over a two-week window and total tree fruit weight (kg tree^-1^) was calculated. Any dropped fruit were added to the total yield after calculating the average individual fruit weight (50 fruit average) and multiplying by the number of dropped fruit.

Fruit samples (total of 5 commercially ripe fruit) were harvested from eight trees per treatment in the replant orchard, and from twelve trees in the virgin orchard. The samples were stored for 24 hours at 2°C, then measured for fruit quality using the methods described by Abdelghafar et al. (2018) in which fruit size, mass, and firmness were measured using a fruit texture analyzer (GÜSS Manufacturing (Pty) Ltd., South Africa), total soluble solids (TSS) were measured by digital refractometry (Atago 3810 PAL-1, Atago, Bellewue, WA, USA) and titratable acidity (% malic acid) was measured by NaOH titration (862 Compact Titrosampler, Metrohm, Riverview, FL, USA). Slices from the same fruit used for fruit texture analysis where then dried at 70°C for 2 weeks, ground to fine powder, and measured for nutrient concentration using the methods for leaf nutrients described previously.

### Statistical analysis

Soil and tree parameters were explored using analysis of variance (ANOVA) comparing the main effects of compost (1x, 2x, and control) and location (replant and virgin) as a 3x2 factorial with orchard row treated as a random effect by year. Soil parameters were additionally explored by soil depth and the factor of year in the model. Significant results of main effects were explored using either Student’s least significant difference or Tukey’s honest significant difference *post hoc* test (α = 0.05). All data were analyzed using the statistical program JMP (Version 14.1.0; SAS Institute, Cary, NC, USA).

## Results

### Soil parameters

**Figure 1:**
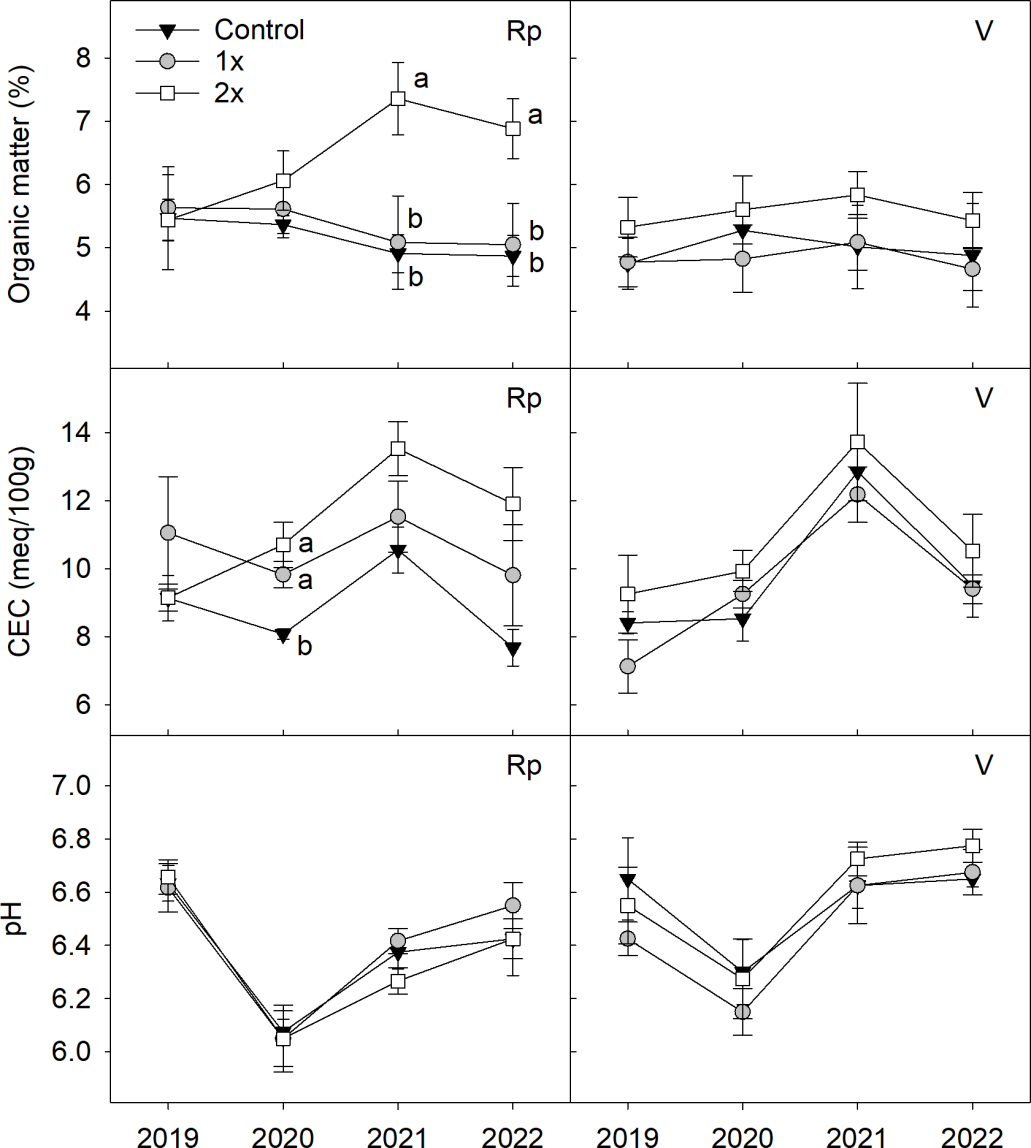
Influence of 1x, 2x, or no (control) compost amendments on organic matter (%), cation exchange capacity (CEC, meq/100g soil), and pH at a 15 cm soil depth in a replant (Rp) or virgin **(V)** peach orchard, 2019-2022 (n = 4-6). Statistical differences between the treatments are shown by letters using Tukey’s honest significant difference mean separation test (α = 0.05) during each year and error bars represent ± standard error of the mean.

OM at 15 cm depth in the 2x compost treatment was higher than the 1x and control treatments by the third (*F* = 5.7, *P* ≤ 0.05) and fourth (*F* = 4.9, *P* ≤ 0.05) year in the replant location, but was similar between all three treatments in the virgin location (**Figure 1**). The control treatment in the replant and virgin locations also had > 4.5% OM content over the study years, despite not receiving any compost. The CEC in the replant location was higher in both the 1x and 2x treatments compared to the control (*F* = 8.8, *P* ≤ 0.01) in 2020, and while the trend continued in 2021 and 2022, there were no differences between the treatments. The CEC in the virgin location was similar between the three treatments during the study years. The soil pH was similar between compost treatments in both orchard locations during the four years of sampling. Bulk density of the 1x and 2x treatments to a depth of 15 cm showed no differences compared to the control soil in either orchard location at the conclusion of the study (*P* > 0.05, data not shown).

At the 15cm depth across the study years and two locations, the compost treatment had a strong effect (*P* ≤ 0.01) on nearly every soil nutrient measured, except for Mg (**Table 2**). A significant effect of compost treatment was less consistent at the 45 cm depth, however both B and Na were higher (*P* ≤ 0.001) within the 2x treatment than the control over the study in both depths. Less difference was found by the factor of orchard location, however addition of OM at both the 1x and 2x rate reduced available soil Cu in the replant location in comparison to the control, while soil Cu was similar between the three treatments in the virgin location at 15 cm depth. The effect of year was the most significant effect for many soil parameters at both 15 and 45 cm depths, but accumulation or depletion trends of soil nutrients were often location specific. There was a strong increase of soil P within the 2x rate in the replant location at 15 cm depth while P decreased over time at the 45 cm depth. Soil Mn also decreased in both orchard locations at the 15 and 45 cm depth.

**Table 2:** Significance of compost treatment (treatment), orchard location (orchard), year of sampling (year), and their interactions on soil nutrients, cation exchange capacity (CEC), pH, and organic matter (OM) at 15 and 45 cm depth.

Soil Depth	Parameter	Soil Nutrient												
		NO_3_	P	K	Ca	Mg	Zn	Mn	Cu	B	Na	CEC	pH	OM
15 cm	Treatment	**	***	***	**		***	**	***	***	***	**	***	***
	Orchard			*		**	*		***				***	*
	Year	**	***		***	*	**	***	***	***	***	***	***	
	Treatment*Orchard		**				*	**	***					
	Treatment*Year		**	*					*					
	Orchard*Year					**							*	
	Treatment*Orchard*Year		*											
45 cm	Treatment	*	*	*		*				***	***	**		
	Orchard	*		**	**	*		**				**	**	
	Year	***	***		*		***	***	***	***	***	***	**	
	Treatment*Orchard												*	
	Treatment*Year	**									**			
	Orchard*Year													
	Treatment*Orchard*Year													

The asterisks *, **, or ***, represent significance at ≤ 0.05. ≤ 0.01, or ≤ 0.001, respectively. Organic matter was not measured at 45 cm depth, while blank cells show no significance (*P* > 0.05).

**Figure 2:**
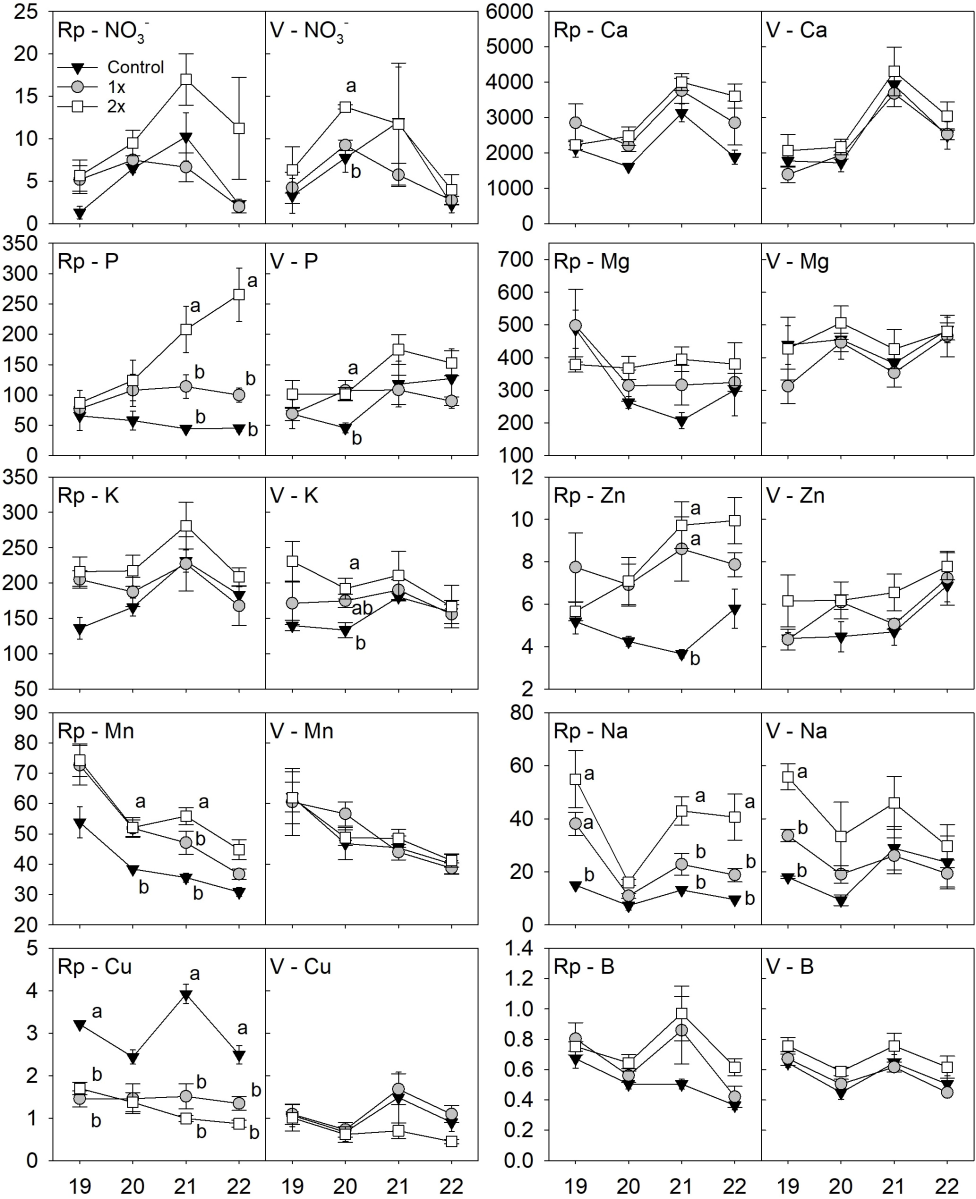
Replant (Rp) or Virgin **(V)** peach orchard soil nutrients including nitrate ( $NO_{3}^{-}$ ), calcium (Ca), phosphorus **(P)**, magnesium (Mg), potassium **(K)**, manganese (Mn), sodium (Na), copper (Cu) and boron **(B)** at 15 cm depth between either 1x, 2x or no compost (Control) from 2019 (19) to 2022 (22). All nutrient concentrations are expressed in kg ha^-1^ but nitrate ( $NO_{3}^{-}$ ), which is expressed in ppm. Statistical differences between the treatments are shown by letters using Tukey’s honest significant difference mean separation test (α = 0.05) during each year and error bars represent ± standard error of the mean.

**Figure 3:**
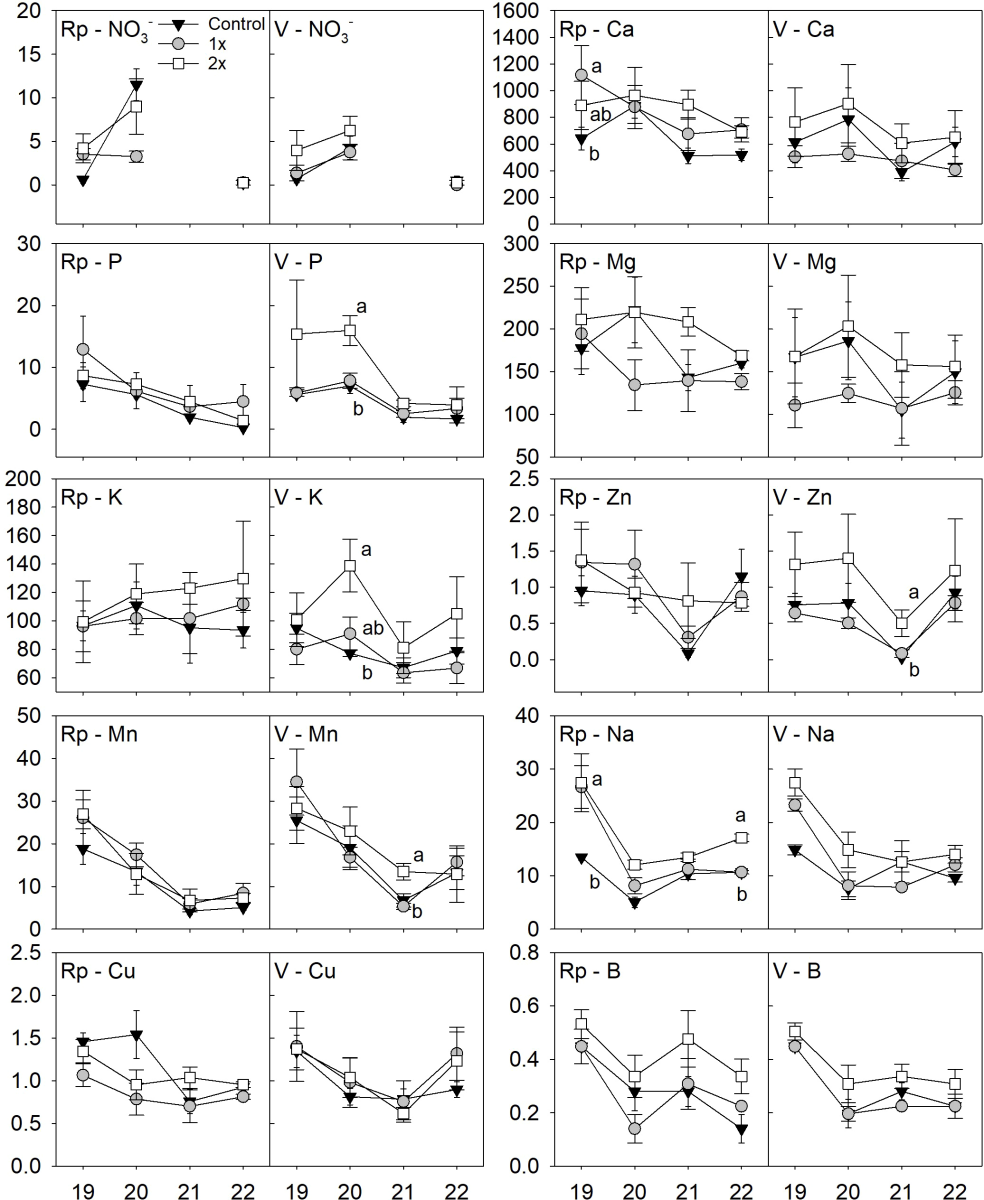
Replant (Rp) or Virgin **(V)** peach orchard soil nutrients including nitrate ( $NO_{3}^{-}$ ), calcium (Ca), phosphorus **(P)**, magnesium (Mg), potassium **(K)**, manganese (Mn), sodium (Na), copper (Cu) and boron **(B)** at 45 cm depth between either 1x, 2x or no compost (Control) from 2019 (19) to 2022 (22). All nutrient concentrations are expressed in kg ha^-1^ but nitrate ( $NO_{3}^{-}$ ), which is expressed in ppm. Statistical differences between the treatments are shown by letters using Tukey’s honest significant difference mean separation test (α = 0.05) during each year and error bars represent ± standard error of the mean.

By year, significant differences between the compost treatments were occasionally observed at 15 cm (**Figure 2**) and 45 cm depths (**Figure 3**) in either the replant or virgin location. In the replant location at 15 cm depth, differences were found for P and micronutrients. Soil P was higher in the 2x compost treatment than the 1x and control in 2021 (*F* = 31.0, *P* ≤ 0.01) and 2022 (*F* = 8.3, *P* ≤ 0.05). Soil Mn was higher in the 1x and 2x rates than the control in 2020 (*F* =18.6, *P* ≤ 0.05), higher in the 2x rate than the 1x and control in 2021 (*F* = 44.0, *P* ≤ 0.01). Soil Cu was higher in the control treatment than the 1x and 2x compost rates in 2019 (*F* = 13.8, *P* ≤ 0.05), 2021 (*F* = 29.7, *P* ≤ 0.05), and 2022 (*F* = 24.2, *P* ≤ 0.05). Soil Zn was also found to be higher in the 1x and 2x rates compared to the control in 2021 (*F* = 68.6, *P* ≤ 0.01) while Na was higher in the 2x and 1x rate than the control in 2019 (*F* = 10.4, *P* ≤ 0.01) and higher in the 2x rate than the 1x and control in 2021 (*F* = 11.9, *P* ≤ 0.01) and 2022 (*F* = 9.3, *P* ≤ 0.01). On the other hand, in the virgin location at 15 cm depth, nitrate ( $NO_{3}^{-}$ ) was significantly higher (*F* = 7.6, *P* ≤ 0.05) during 2020 in the 2x rate compared to the 1x and control. Soil P was higher in the 1x and 2x compared to the control (*F* = 27.8, *P* ≤ 0.01) during 2020 and K was higher (*F* = 5.2, *P* ≤ 0.05) in the 2x rate than the control in 2020. Soil Na was higher in the 2x rate than the 1x and control treatments during the spring of 2019 (*F* = 24.7, *P* ≤ 0.001).

Yearly differences between compost treatments were also observed at 45 cm depth. In the replant location, Ca was higher in the 1x treatment than the control (*F* = 11.2, *P* ≤ 0.05) in 2019. Na was higher in the 1x and 2x rates compared to the control in 2019 (*F* = 16.4, *P* ≤ 0.05) while the 2x treatment was higher than the 1x and control in 2022 (*F* = 14.2, *P* ≤ 0.05). In the virgin location at 45 cm depth, P was higher in the 2x treatment compared to 1x and control (*F* = 8.9, *P* ≤ 0.05) and K was higher (*F* = 5.4, *P* ≤ 0.05) in the 2x compared to the control in 2020. In 2021, the 2x treatment was higher than 1x and control for Mn (*F* = 6.0, *P* ≤ 0.05) and Zn (*F* = 6.4, *P* ≤ 0.05).

### Soil moisture tension and stem water potential

**Figure 4:**
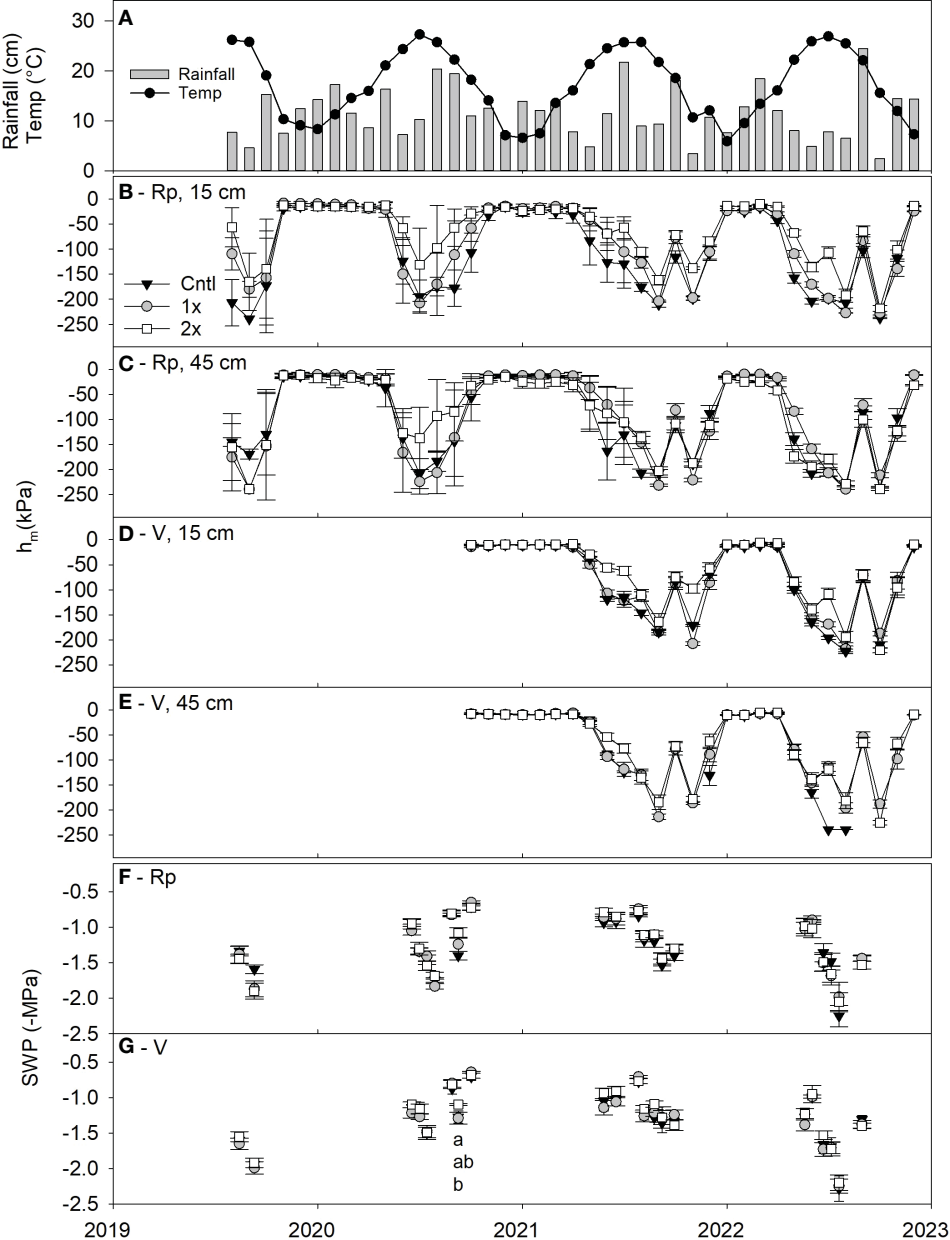
Peach orchard **(A)** monthly rainfall (grey bars, cm) and temperature (black circle line, Temp, °C) along with average monthly soil moisture tension (h_m_, kPa, n = 28-31) in the replant location **(B)** at 15 cm and **(C)** 45 cm depth; the virgin orchard location **(D)** at 15 cm and **(E)** 45 cm depth; and stem water potential (SWP, -MPa, n = 6) in the replant location **(F)** and the virgin location **(G)** between either 1x, 2x or no compost (Cntl) treatments from 2019 to 2022. Statistical differences between treatments of the SWP measurements are shown by letters using Tukey’s honest significant difference mean separation test (α = 0.05) and error bars represent ± standard error of the mean.

Soil moisture at both 15 cm and 45 cm depths generally followed seasonal rainfall patterns (**Figure 4A**), having lower h_m_ during the winter months and larger h_m_ during the growing season. During the winter months and leading into the growing season (December-April), all treatments were similar statistically, but the trend of 2x compost rate appeared to have lower h_m_ at both the 15 and 45 cm depths than the 1x and control during the growing season of 2020 in the replant location (**Figures 4B, C**). Sensors in the virgin location showed the same trend in 2021 and 2022 as the 2x rate often had statistically similar, but lower h_m_ compared to the control treatment during the spring season at both soil depths (**Figures 4D, E**).

Measurements of SWP also appeared to follow seasonal moisture trends during each growing season, but few statistical differences were observed between the three amendment treatments in either orchard location (**Figures 4F, G**). Only a single date in 2020 showed a difference between the three treatments, where 2x trees had less negative SWP *(F* = 4.1, *P* ≤ 0.05) compared to the control trees in the virgin location (**Figure 4G**). Over the entire growing season across compost treatments, the virgin orchard had lower SWP than the replant location in 2019 (*F* = 6.7, *P* ≤ 0.05). No differences were observed in 2020, 2021 or 2022.

### Tree biomass

By the third growing season in 2021, TCSA and canopy width were larger for the 2x compost treatment compared to the control, but only in the virgin location (**Table 3**). Across treatments, TCSA was higher in the virgin location than the replant location in 2020 (*F* = 10.0, *P* ≤ 0.002), but similar between the two locations all other years across the compost treatments. By the final year of the study in 2022, after receiving no additional synthetic fertilizer in 2020, the 2x treatment had higher TCSA (*F* = 4.1, *P* ≤ 0.05) than the 1x or control trees across the two orchard locations. Within each orchard location, the virgin location had higher TCSA in the 2x rate compared to the control in 2021 (*F* = 4.6, *P* ≤ 0.05) and 2022 (*F* = 3.3, *P* ≤ 0.05). Measurements of canopy volume were not different by compost treatment in either orchard location in 2020, but the virgin location had larger tree volume within the 2x compost rate compared to the control in 2021 (*F* = 4.0, *P* ≤ 0.05) and 2022 (*F* = 3.3, *P* ≤ 0.05). Across compost treatments, canopy volume was larger in the virgin location in comparison to the replant orchard in 2020 (*F* = 11.5, *P* ≤ 0.001), 2021 (*F* = 8.6, *P* ≤ 0.01), and 2022 (*F* = 6.5, *P* ≤ 0.05).

**Table 3:** Average trunk cross sectional area (TCSA, cm^2^) and average canopy volume (m^2^) during the growing season between peach trees established on a replant (n = 16) or virgin orchard location (n = 24) amended annually with either 1x, 2x, or no (control) compost.

Year	Measurement	Virgin			Replant		
		Control	1x	2x	Control	1x	2x
2019	TCSA	9.1	9.3	9.4	8.7	9.2	9.1
	Canopy volume	–	–	–	–	–	–
2020	TCSA	49.6	51	49.8	47.9	45.2	47.4
	Canopy volume	9.6	10.4	11.2	8.4	7.2	9.5
2021	TCSA	77.9 b	80.2 ab	83.3 a	79.3	81.4	80.9
	Canopy volume	24.2 b	26.9 ab	29.8 a	23.8	19.9	25.0
2022	TCSA	102.8 b	103.5 b	110.2 a	101.4	100.1	105.6
	Canopy volume	33.6 b	38.1 ab	40.0 a	33.3	30.2	34.6

Different letters show significant differences between treatments within each orchard using Student’s least significant difference mean separation test (*P* ≤ 0.05).

### Tree leaf nutrients

**Figure 5:**
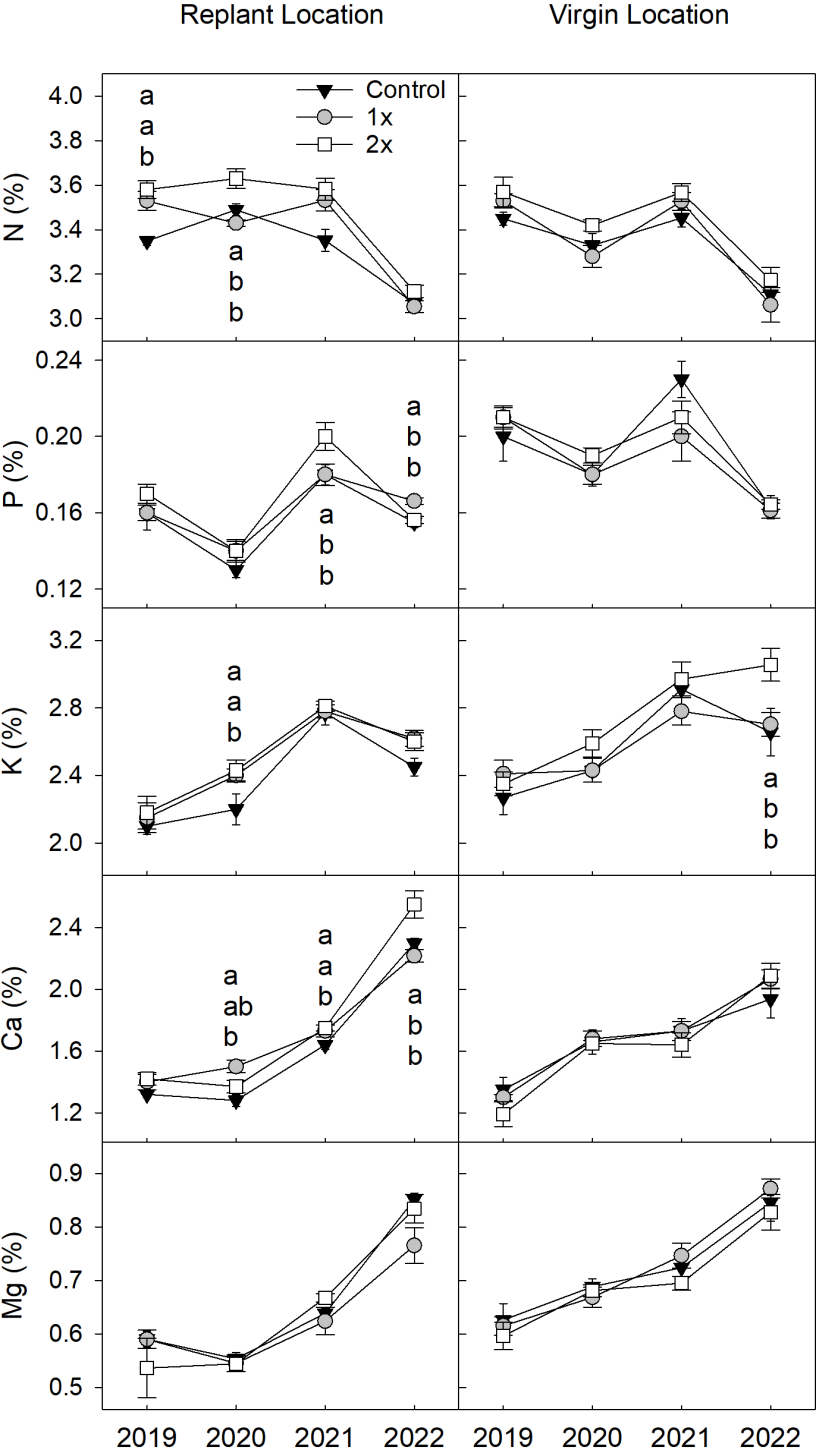
Leaf nutrient concentrations (%) of nitrogen **(N)**, phosphorus **(P)**, potassium **(K)**, calcium (Ca), and magnesium (Mg) between peach trees grown with either 1x, 2x, or no compost (control) amendment added annually during each year of study, 2019-2022 (n = 6). Different letters show significant differences using Tukey’s honest significant difference mean separation test (α = 0.05), error bars represent ± standard error of the mean.

Trees in both locations showed an increase of leaf K, Ca and Mg concentration while maintaining similar leaf N and P over the four years of study (**Figure 5**). Nearly all annual nutrient differences between the compost treatments occurred in the replant location. In 2019, N was higher within the 2x treatment compared to the control (*F* = 11.9, *P* ≤ 0.01). In 2020, N was higher in the 2x treatment leaves compared to the 1x and control treatment trees (*F* = 11.7, *P* ≤ 0.01), K was higher in both 1x and 2x treatments compared to the control (*F* = 4.1, *P* ≤ 0.05), and Ca was higher for the 1x treatment compared to the control (*F* = 3.2, *P* ≤ 0.05). In 2021, the 2x treatment had higher P (*F* = 4.4, *P* ≤ 0.05) and Ca (*F* = 4.0, *P* ≤ 0.05) compared to the control. In 2022, the 1x treatment had higher P (*F* = 8.3, *P* ≤ 0.01) than both 2x and control while the 2x treatment had higher Ca (*F* = 8.8, *P* ≤ 0.01) than the 1x and control. The only difference found any year of the study between the compost treatments in the virgin location occurred in 2022, when K was higher (*F* = 4.2, *P<* 0.05) in the 2x compost rate compared to the control.

Several differences of leaf nutrients were observed between the compost treatments across orchard locations. In 2020, leaf K (*F* = 3.4, *P* ≤ 0.05) was higher in 2x compared to control and Ca (*F* = 3.8, *P* ≤ 0.05) was higher within 1x compared to the control across orchard locations. Across orchard locations in 2019 and 2021, there were no differences in K between compost treatments. In 2022, the 2x compost increased leaf K (*F* = 5.48, *P* ≤ 0.01) and Ca (*F* = 4.0, *P* ≤ 0.05) across orchard locations compared to the control. The main effect of orchard location had a stronger influence on leaf nutrient concentration, as Ca was higher in the replant location (*F* = 4.8, *P* ≤ 0.05) and P was higher (*F* = 64.3, *P* ≤ 0.0001) in the virgin location across compost treatments in 2019. In 2020, the virgin location had higher leaf K (*F* = 5.6, *P* ≤ 0.05), Ca (*F* = 50.4, *P* ≤ 0.0001), Mg (*F* = 171.1, *P* ≤ 0.0001) and P (*F* =136.8, *P* ≤ 0.0001) than the replant location, while concentrations of N (*F* = 24.6, *P* ≤ 0.0001) in the replant location were higher than in the virgin location. In 2021, the virgin location had higher Mg (*F* = 28.4, *P* ≤ 0.0001) and P (*F* = 8.3, *P* ≤ 0.01) than the replant location. In 2022, K was lower (*F* = 13.2, P< 0.001) and Ca was higher (*F* = 26.7, *P* ≤ 0.001) in the replant location compared to the virgin location.

### Fruit nutrients, yield and quality

In both 2021 and 2022, fruit from both orchard locations were harvested and analyzed for nutrients, but the compost treatments did not have any effect on fruit nutrients within either orchard location (data not shown). In 2021, the virgin location had higher concentration of fruit P (*F* = 8.7, *P* ≤ 0.01), K (*F* = 15.3, *P* ≤ 0.001), Ca (*F* = 21.8, *P ≤* 0.0001), and Mg (*F* = 29.0, *P* ≤ 0.0001) compared to the replant location across the compost treatments. Higher concentrations of fruit Mg (*F* = 14.3, *P* ≤ 0.01) and P (*F* =10.8, *P* ≤ 0.01) were found in the virgin location fruit compared to the replant location fruit across the compost treatments. No differences between fruit N were found between the compost treatments either year, while fruit had generally less N, P, K, and Mg in 2022 compared to 2021.

The 2x compost treatment increased fruit yield (*F* = 6.3, *P ≤* 0.01) compared to the control in the virgin location in 2022, but fruit mass, size, TSS and acidity were similar between treatments in the virgin and replant location during the two years of fruit harvest (**Table 4**). In 2021, fruit firmness was higher (*F* = 4.5, *P ≤* 0.05) within the control treatment compared to the 2x compost rate in the virgin location. Across the compost treatments in 2021, fruit had greater firmness (*F* = 45.8, *P≤* 0.0001) and size (*F* = 14.5, *P≤* 0.001) in the virgin location than the replant location. Fruit in the replant location had greater TSS (*F* = 17.4, *P≤* 0.001) and less acidity (*F* = 32.3, *P≤* 0.001) compared to the virgin location. In 2022, there was a significant interaction between compost treatment and orchard location in regard to fruit firmness, where fruit in the virgin location had similar firmness, while 2x fruit were firmer than control fruit (*F* = 5.7, *P ≤* 0.05). Additionally in 2022, acidity was lower in the replant location (*F* = 10.1, *P ≤* 0.01) than the virgin location across the compost treatments.

**Table 4:** Total yield (kg tree^-1^), mass (g), size (diameter, cm), firmness (kg cm^-2^), total soluble solids (TSS, %), and titratable acidity (as malic acid, acidity, %) of peach fruit from trees grown on either new land (virgin) or a replant site and between 1x, 2x, or no (control) compost amendment.

Year	Parameter	Location					
		*Virgin*			*Replant*		
		Control	1x	2x	Control	1x	2x
2021	Yield	28.8	31.3	33.9	35.2	30.9	34.9
	Mass	252.2	239.5	252.3	240.8	240.4	247.2
	Size	8.1	8.0	8.2	7.9	7.9	8.0
	Firmness	4.6 a	3.9 ab	3.8 b	2.9	3.0	2.6
	TSS	11.6	11.5	11.5	12.0	12.4	12.6
	Acidity	0.62	0.59	0.58	0.52	0.53	0.51
2022	Yield	61.6 b	68.8 ab	79.4 a	75.0	69.2	74.9
	Mass	226.2	218.9	219.1	222.9	223.6	213.5
	Size	7.9	7.8	7.8	7.9	7.9	7.8
	Firmness	4.6	4.8	4.1	3.0	3.5	4.1
	TSS	12.7	12.2	11.1	13.2	13.3	12.1
	Acidity	0.69	0.71	0.66	0.52	0.63	0.62

Different letters show significant differences between treatments by location using Tukey’s honest significant difference mean separation test (n = 30).

## Discussion

The results show that the application of food waste compost, mixed into the soil before planting and subsequent topical additions annually reduced or replaced spring synthetic fertilizer input throughout the first four years of orchard establishment, and had similar, if not improved, vegetative growth in comparison to the standard management practices. The site conditions and historical management may also contribute to the utility and ecosystem services of amendments (De Leijster et al., 2019) since compost applications increased OM in the replant location, but not the virgin location compared to the control. Nevertheless, increasing OM in the replant location after only several years agrees with other studies which have observed increased soil organic carbon after adding OM amendments to the soil surface (Villa et al., 2021; Khalsa et al., 2022). On the other hand, more than four years may be necessary to observe changes to OM or tree parameters when using amendments (Neilsen et al., 2014; Toselli et al., 2019) and the four-year duration of this study is insufficient to understand the treatment effects over the lifetime of the orchard.

### Tree biomass

By the fourth year of the experiment, the TCSA of 2x compost trees was larger than the control trees across orchard locations, even after the 2x treatment received no synthetic fertilizer in 2020. Annual additions of compost provided the N needed to grow similar tree biomass during the first 14 years of growth in Italy (Toselli et al., 2019), while other resources including available N and soil moisture may better correlate with trunk size (Reeve et al., 2017). Since both the virgin and replant locations had sufficient summer leaf N throughout our study and similar amount of N within shoot tissue during dormancy (data not shown), soil moisture may have been more consequential than N in creating larger TCSA and canopy volume for the 2x treatment compared to the control trees. As no significant differences were observed in fruit yield, whether larger TCSA or canopy volume will generate larger marketable yields of the lifetime of the trees will require additional years of study.

### Fruit yield, nutrients, and quality

Very few differences were observed between the compost treatments for fruit parameters measured in the current study, but this is similar to other fruit orchards where the effect of soil amendments have been studied. Regarding yield, integrated systems which added compost to apple trees while using synthetic fertilizer had similar yields compared to conventional and organic methods in Washington (Reganold et al., 2001). Increasing OM through a leguminous cover crop or bark mulch also did not increase cumulative yield over two years in Canada (Neilsen et al., 2014). In our study, the virgin orchard 2x compost trees had higher yield in comparison to the virgin control trees in 2022. While yield measurements of compost-amended nectarine trees in the Mediterranean Basin have shown higher yields over a single year compared to control trees (Baldi et al., 2010b), additional yield and nutrient analysis will be required to justify long-term use of compost in the southeastern U.S. Long-term studies on nectarines in Italy and apples in New York show similar cumulative yield results from elevated OM after applying compost or mulch, respectively, compared to standard management using mineral fertilizer (Atucha et al., 2011; Toselli et al., 2019). Differences in fruit nutrients and quality have been observed in previous studies as a consequence of adding OM. Scientists in Brazil found a significant increase of peach fruit N content correlated to increasing amounts of organic compost, but found no differences in other nutrients, TSS, or acidity (Melo et al., 2016). Alternatively, fruit size and TSS content increased after compost application compared to mineral fertilizer in pear fruit (Sorrenti et al., 2012), and a high rate of compost has been shown to increase nectarine fruit firmness as a result of delayed maturation from increased N availability in Italy (Toselli et al., 2019). In our experiment, the 2x treatment appeared to delay fruit maturation in 2022. Since the 2022 compost analysis showed much higher total N than previous years, available N may have caused this delay but did not change other fruit quality parameters measured. Optimizing $NO_{3}^{-}$ levels and preventing N loss within orchards can be challenging due to the perennial nature of trees and ongoing mineralization of compost-derived N may have exceeded tree demand, leading to a delay (Weinbaum et al., 1992; Cui et al., 2020). In addition to a possible nutrient effect, the firmness of 2x compost fruit were found to be statistically lower than the control in the virgin location in 2021, and the virgin location had firmer fruit than the replant location across compost treatments both years. Since leaf and shoot N, along with fruit Ca were similar between the two orchard locations, other factors including sunlight exposure due to the virgin location east-facing azimuth or higher temperature in the replant location may explain differences in fruit firmness (Layne et al., 2001), although neither was measured. Regardless, the replant location fruit firmness was not unusually soft and was similar to readings (2.5-3.6 kg cm^-2^) observed from other cultivars at the same farm over three years (Gasic et al., 2015).

### Tree nutrient status, soil OM, and soil nutrients

Nutrient status of leaf tissue for the 1x and 2x compost treatments maintained sufficient levels throughout the study or similar levels to that of the control treatment despite a reduction of the recommended synthetic fertilizer rate during the spring (Blaauw et al., 2021). These results are similar to other compost studies in Italy and Brazil which were able to replace synthetic fertilizers and had similar leaf nutrient status compared to control trees (Baldi et al., 2010b; Melo et al., 2016). However, soil analysis and leaf analysis did not always seem to match. Soil analysis showed no differences to soil Ca by treatment, but higher leaf Ca was present by the third and fourth season for the 2x treatment. Alternatively, despite having similar pH between treatments but higher amounts of soil P at the 15 cm depth in the 2x treatment, leaf P in the replant location was similar across time between the compost treatments, suggesting that tree status cannot always be predicted by soil analysis (Robinson, 1980). Analysis of other plant organs, known to have different amounts of perennial nutrient concentrations such as roots (El-Jendoubi et al., 2013), may provide additional understanding of how OM influence nutrient storage during dormancy. Although no roots were studied, young peach trees amended with compost previously have shown higher root production in comparison to mineral fertilizer (Baldi et al., 2010a).

The increase of soil OM within the replant orchard is a result of both the compost applied and the methodology to test the soil. Randomly selected locations for soil sampling included compost which had decomposed onto the soil surface. Unlike other studies which have incorporated OM into the orchard using light tillage (Sorrenti et al., 2012; Baldi et al., 2016), the surface applications of compost after the initial planting year provided little soil carbon to deeper soil layers, but still resulted in increased OM percentages in the replant location over time. Peach roots were never observed growing through the compost applied to the surface, and the compost material on the surface often appeared dry throughout the growing season. Regardless, trees still received sufficient mineralized nutrients from the compost, as synthetic fertilizer was either reduced by 80% or 100% during the spring when the 1x and 2x compost rates were applied, respectively. Since both orchards were the same soil type, differences in OM accumulation over the study period may be due to prior orchard use, environmental conditions, and existing soil microbiology between the two sites. The virgin location was east facing and downhill from the replant location and may have had greater moisture availability for microorganisms, which potentially hastened the decomposition and mineralization of applied OM over the growing season (Zornoza et al., 2018). Despite the differences between the two locations, both locations had abnormally high OM regardless of compost treatment, as previous reported values are lower at the research farm, normally between 1.5% and 3%. Insufficient drying of the soil samples prior to loss on ignition testing may also partially explain the elevated percentages. Regardless of the procedure followed, surface residues were removed prior to taking the soil samples, but the soil taken from the first 15 cm of soil may have had a large amount of organic matter from plant debris mixed into them following berm creation from the fields prior to discing. In a similar way, similarities observed between the bulk density regardless of treatment suggests that surface application of OM may not quickly change physical properties of lower soil depths.

Soil nutrients were also influenced by compost treatments over the study years. Soil N can accumulate over time after repeated compost additions (Toselli et al., 2019), but $NO_{3}^{-}$ analysis over four years did not show a consistent trend of increase for either 1x or 2x compost rates in comparison to the control at any depth. Without measuring during multiple times during the growing season or other forms of N, such as ammonium within the soil (Baldi et al., 2010b), this study may have underestimated the amount of N within the compost treatments given there was evidence of a N effect on TCSA and a delay of fruit maturation in 2022. Soils amended with OM often have higher P and K than soils without compost (Baldi et al., 2010b; Neilsen et al., 2014) but the current study showed no significant increase of K in either location and the virgin location had stable values of soil P at 15 cm depth. Regardless, the increase of P in the replant orchard at 15 cm depth should be monitored in future years as it may lead to some environmental risks through leaching or runoff (Preusch & Tworkoski, 2003). Higher soil P and K within the compost treatments did not consistently reflect higher leaf P and K, highlighting a need to better correlate soil status with plant status to meet sustainability goals.

Different soil metals were also influenced by compost treatments. Another factor could be residual copper from previous pest management in the replant orchard, which may have slowed OM decomposition (Sauvé et al., 1997) compared to the virgin location; at the same time, the bioavailability of Cu was greatly reduced by both compost rates and the bioremediation from OM could improve soil microbiology and macrofauna, which normally have reduced populations and diversity under high Cu soils (Centofanti et al., 2016; Sofo et al., 2020). Soil Mn was observed to decrease over time in both orchards at both soil depths, although higher amounts of Mn were applied within the compost each year. Less soil disturbance and higher OM content are known to increase the availability of Mn in agronomic crops (Moreira et al., 2016), therefore the decrease of Mn over time during soil testing was unexpected. The increase of soil Na could also be a negative consequence of the compost material, but studies have shown that increasing OM can improve salt affected soils in semi-arid environments (Garcia-Franco et al., 2021) and further study is needed to understand whether certain metals accumulate over time in specific regions. Provided quality compost material, it is possible that no accumulation or excessive amount of soil nutrient may occur following annual additions of OM (Baldi et al., 2014).

### Soil moisture tension and stem water potential

The 2x application of compost seemed to improve soil moisture at both the 15 and 45 cm depth during the growing season. The increased infiltration of water was probably due to a reduction of soil crusting and the compost acted similarly to a mulch within the orchard, preserving soil moisture by reducing evaporation (Campi et al., 2020) in comparison to the control. Other research performed in fruit and nut orchards have observed similar results. For instance, topical application of green waste and manure wood chip compost in almond orchards increased available water compared to an unamended control in loamy soil over two years (Villa et al., 2021) and the growth of pecan trees was increased in part due to improved soil moisture conditions after applying hardwood mulch (Smith et al., 2000). Additions of straw mulch have also been shown to improve water infiltration and slow water movement in comparison to bare soil (Keesstra et al., 2019). Improving water status during orchard establishment could prove beneficial for growers, especially those growers that do not irrigate trees prior to when fruit production occurs, as improving tree water status during the early years increase young tree TCSA, canopy volume, and initial yield (Casamali et al., 2021). Although buffered soil moisture conditions may account for larger TCSA in the 2x compost rate, additions of compost largely did not change SWP during the growing season, except for a single point in 2020 when the 2x rate had less water stress than the control. These results are similar to a study of young almond trees in California, which were observed to have numerically lower SWP measurements over two growing seasons after applications of composted dairy manure (Lepsch et al., 2019) but were not significantly different than control trees without OM applications. Understanding water dynamics within soils amended with OM in humid climates will require further research, especially regarding water redistribution events, infiltration, and evaporation rates as well as root growth patterns.

### Compost on the orchard ecosystem

A management style in different peach growing regions includes planting young peaches atop soil berms. In the present study, the berms of both orchards created a unique surface topography in which applied compost eroded from the sides of the berms and was retained along the base. The creation of berms further complicates how regional growers may manage orchard soils as channelized water along the berms can increase erosion rates, suggesting the practice may not be environmentally sustainable in the long-term (Atucha et al., 2013; Keesstra et al., 2019). Tree rows void of any substantial surface cover following herbicide sprays experience higher soil and water losses (Keesstra et al., 2016), and it was observed the berms of the replant and virgin locations channelized water regardless of compost treatment. Application of cover, such as mulch, to the surface can slow the rate of nutrient and water runoff by potentially improving soil structure and increasing water infiltration rates, but additional studies should address the impact of berms on the orchard ecosystem, especially when combined with alternative management practices which increase soil OM.

## Conclusions

The 1x and 2x compost rates, which replaced 80% or 100% of the spring fertilizer amount, respectively, resulted in similar leaf nutrient content, fruit yield, and fruit quality in both orchard locations. Although adding compost did not increase yield by the fourth year in comparison to trees without compost, completely eliminating fertilizer from the 2x compost treatment in 2020 did not appear to have any negative consequences on growth as the 2x treatment yielded larger TCSA and canopy volume by the end of the study across both orchard locations. Changes in soil parameters were dependent on orchard site, as soil OM, P, and Zn increased, and Cu decreased after applications of compost in the replant location. The compost additions had a more pronounced effect upon measured soil parameters in the replant site, but only the virgin location showed difference in tree growth or yield. Although other growing regions have reported an ability to replace synthetic fertilizers and improve various ecosystem services including nutrient cycling using compost, additional research is required within the subtropical southeastern context to explore the effect of compost applied to the soil surface over the lifetime of an orchard, including potential changes to soil biology or major pests and diseases, and to determine the economic sustainability within the region.

## Data availability statement

The original contributions presented in the study are included in the article/**Supplementary Material**. Further inquiries can be directed to the corresponding author.

## Author contributions

BL and JM planned the experiment together and BL carried out data collection and analysis. BL wrote the manuscript with support from JM. Both authors contributed to the article and approved the submitted version.
