# Supplementary material for: Annual compost amendments can replace synthetic fertilizer, improve soil moisture, and ensure tree performance during peach orchard establishment in a humid subtropical climate
Source: Front Plant Sci. 2023 May 8;14:1172038. doi: 10.3389/fpls.2023.1172038 (PMC10200951; doi:10.3389/fpls.2023.1172038)
Supplement: Supplementary file 1 [file Table_1.docx]

**Table S1.** Average chemical characteristics (g kg^-1^ dry weight), C:N ratio, organic matter (%), EC (dS m^-1^), and pH of the food waste compost used during the study years of 2019-2022.

| **Compost characteristic** | 2019 | 2020 | 2021 | 2022 |
| --- | --- | --- | --- | --- |
| Ammonium nitrogen | 1.00 | 0.00 | 0.10 | 0.10 |
| Total nitrogen | 12.27 | 10.12 | 8.27 | 23.02 |
| Phosphorus (P_2_O_5_) | 3.44 | 3.74 | 4.51 | 11.22 |
| Potassium (K_2_O) | 4.17 | 1.64 | 2.75 | 6.43 |
| Calcium | 6.55 | 6.35 | 8.54 | 18.47 |
| Magnesium | 1.04 | 1.00 | 1.38 | 2.62 |
| Sulfur | 0.96 | 0.86 | 0.82 | 2.80 |
| Zinc | 0.03 | 0.03 | 0.04 | 0.09 |
| Copper | 0.01 | 0.01 | 0.02 | 0.02 |
| Manganese | 0.01 | 0.14 | 0.16 | 0.37 |
| Iron | 3.82 | 4.50 | 5.14 | 9.68 |
| Sodium | 2.08 | 0.22 | 0.71 | 2.22 |
| Aluminum | 4.38 | 5.12 | 5.70 | 11.15 |
|  |  |  |  |  |
| C:N | 5.68 | 12.25 | 11.72 | 13.04 |
| Organic Matter | 26.50 | 39.00 | 19.40 | 41.30 |
| EC | 4.53 | 0.30 | 0.63 | 0.74 |
| pH | 8.20 | 6.60 | 7.50 | 7.10 |
